# Mechanism of Surface Defects in Ultra-Precision Machining of Sesquioxide Laser Crystal Tm: GdScO_3_

**DOI:** 10.3390/mi13081250

**Published:** 2022-08-03

**Authors:** Yuanyuan Fang, Hongbo He, Aihuan Dun, Long Zhang

**Affiliations:** 1 Shanghai Institute of Optics and Fine Mechanics, Chinese Academy of Sciences, Shanghai 201800, China; hbhe@siom.ac.cn (H.H.); dunaihuan0810@163.com (A.D.); lzhang@siom.ac.cn (L.Z.); 2 University of Chinese Academy of Sciences, Beijing 100049, China

**Keywords:** sesquioxide laser crystal, surface defect, MD simulation

## Abstract

It is well-known that the surface quality of laser gain crystal elements is very high in order to ensure the stability of laser system and laser output quality. In the ultra-precision machining process of a new sesquioxide laser crystal Tm: GdScO_3_, it is required to achieve very high surface shape and very low surface defects. In this paper, the molecular dynamics simulation model of single particle grinding was established. It was found that the normal load and tangential friction imposed by abrasive particles on the surface of components cause the spalling of atoms on the substrate surface, which constitutes the removal of materials at the macro-level. At the same time, it causes the displacement of the sub surface atoms, which constitutes the microscopic defects in the structure. Through the structural characterization of macro defects, it was confirmed that the essence of micro defects is the amorphous and distortion of surface structure, and the depth can reach 100 nm. The results of lapping and polishing experiments show that the adjustment of pressure has a limited effect on the improvement of surface defects in the process of machining crystal elements with granular abrasive.

## 1. Introduction

High power laser has been widely used in military, industrial, scientific research, medical, and many other scientific and technological fields because of its high efficiency and high power. In general, YAG crystals or ceramics doped with rare earth (Re) ions are considered to be excellent materials for high-power lasers because of their high conductivity, excellent optical and laser properties [1,2]. In recent years, high-temperature rare earth oxides such as Lu_2_O_3_, Y_2_O_3_, and Sc_2_O_3_ have been considered as potential materials for high-power lasers, because these materials have higher thermal conductivity and lower phonon energy compared with YAG, which determines that these materials can be used for higher power lasers more efficiently [3].

In recent years, GdScO_3_ crystal has attracted the extensive attention of researchers because of its low phonon energy and high thermal stability [4]. GdScO_3_ belongs to the orthorhombic system with the space group of *Pnma* [5]. As well, perovskite structure is well-known for its high tolerance to structural distortion and for allowing different ions to replace at all cation positions [6].

Among many rare earth doped ions, the Tm^3+^ would become the outstanding candidate because of the four-energy-level system of the ^3^H_4_ → ^3^F_4_ transition (~1.5 μm) and ^3^H_4_ → ^3^H_5_ transition (~2.3 μm). So far, different crystalline hosts doped with Tm^3+^ have been demonstrated. Qiu Li [7] successfully prepared Tm^3+^ doped GdScO_3_ crystal by the EFG method. The optical properties and Jo analysis were discussed, and the results showed that Tm: GdScO_3_ has good spectral characteristics and may be a promising material for near-infrared laser operation. Shanming Li [8] successfully prepared Tm^3+^ doped GdScO_3_ crystal by Czochralski (CZ) method for the first time. The polarization absorption and emission characteristics were studied in detail, which demonstrated 2 μm continuous wave laser operation. All results showed that Tm: GdScO_3_ crystal acts as a broadband tunable and ultrashort laser at 2 μm is a promising candidate.

In order to obtain high-quality laser components, in addition to producing high-quality crystals, ultra-precision processing means such as grinding and polishing are also needed to ensure the high integrity of finished components. However, GdScO_3_ laser crystal has the characteristics of high brittleness, close breaking strength and yield strength, and high hardness [9,10,11]. These characteristics will lead to large surface defects and serious sub-surface damage in the processing process, which will seriously affect the performance and life of laser systems [12]. Although there are some processing methods for hard and brittle materials, they are generally in the experimental stage, and the pertinence is not high, especially for the processing of high-temperature rare earth oxide crystal components. Therefore, it is of great significance to study the defect generation and material removal mechanism of oxide laser crystal materials in ultra-precision machining [13].

At present, the processing methods for this kind of laser crystal mainly include cutting, grinding, and polishing. McKay of the University of California carried out the chemical mechanical polishing experiment of YAG single crystal and obtained a super-smooth surface with a surface roughness of 0.2 nm [14]. Ross of the University of Florida in the United States carried out the magnetic field-assisted polishing experiment of YAG and obtained a smooth surface with a surface roughness value of 1 nm [15,16]. The domestic research on this kind of laser crystal mainly focuses on the polishing process. Li Jun, Nanjing University of Aeronautics and Astronautics, etc. processed neodymium-doped yttrium aluminum garnet by chemical mechanical polishing, and the surface roughness value of the component is less than 0.2 nm RMS [17].

Although chemical mechanical polishing can be used to process the ultra-smooth surface of this kind of material, the processing of rare earth oxide laser crystals basically focuses on the polishing process experiment, and there is a lack of research on the ultra-precision machining mechanism of this kind of materials. The material removal mechanism and defect generation mechanism of this kind of material in ultra-precision machining scale are not clear.

The technologies including finite element analysis and molecular dynamics simulation are widely used in the machining modeling of hard and brittle materials. They are important means to study the mechanism of various complex grinding processes [18]. For example, Zhu et al. used finite element analysis to study the initiation and propagation of a single crack in silicon carbide grinding under the maximum undeformed chip thickness. The results show that in the plastic state, the wheel speed has no obvious effect on the element’s surface morphology, but in the brittle state, the element’s surface integrity depends on the wheel speed [19]. Liu et al. used the finite element simulation technology to simulate the SiC indentation and meshing process, and studied the generation and propagation of cracks under different wheel speeds. The results show that high-speed grinding can obtain high material removal rate and good surface and sub-surface quality [20]. However, this sub-micron scale finite element analysis method can be used to simulate the macro mechanical behavior in the process of material processing. For the micro mechanical behaviors such as crystal defects (phase transition, crystallization, dislocation, etc.) and material deformation during machining, the molecular dynamics simulation based on the nano-micro scale can better describe them [21].

## 2. Experimental Devices and Conditions

Firstly, use China’s Shanghai Hui-sheng j5075/ZF inner circle cutting machine to cut the blank crystal into the required shape, and leave a certain machining size allowance, with the size of 32 mm × 32 mm × 2 mm. The whole process of crystal precision machining includes grinding, rough polishing, and fine polishing. Gradually select emery bulk abrasives with particle sizes of 40, 20, and 10 microns for grinding. Then use a particle size of 3 μm diamond powder combined with resin copper disk for rough polishing. After rough polishing, the particle size is 0.1 μm alumina polishing powder combined with asphalt disc for precision polishing to improve surface finish and surface shape accuracy (Grinding and polishing accessories are purchased from UNIVERSAL PHOTONIC, INC., Central Islip, NY, USA).

Japan’s Nikon eclipse e400 pol optical microscope was used to observe the macroscopic damage on the crystal surface after each processing process. Zygo new view8050, USA, white light profilometer was used to measure the surface roughness of the crystal after each process, and the surface micro damage was observed and analyzed. The surface defect morphology was observed by dark field scattered light imaging. Every time the grinding auxiliary material or polishing powder is replaced, the sample needs to be cleaned with ultrasonic waves to avoid polluting and damaging the surface. For the regions with surface defects, samples for transmission electron microscope (FEI Talos F200X, Waltham, MA, USA) observation were prepared by a focused ion beam electron microscope. In order to study the changes in surface structure during mechanochemical wear, we used reactive force field (ReaxFF) for molecular dynamics simulation. The elastic modulus of the crystal was measured and analyzed by using the UMS-200 advanced ultrasonic echo material characterization system of TECLAB China. The scratch test was carried out with Germany’s Bruker UMT TRIBOLAB nano scratch instrument, which increased from 0 to a constant load of 100 mN, 200 mN, and 300 mN respectively, and the scratch speed was 1 μm/s, and the sliding distance is set to 50 μm. It is carried out in a clean room with a room temperature of 21 °C and humidity of 45%.

## 3. Results and Discussion

### 3.1. Surface Defect Morphology in Different Process

**Figure 1:**
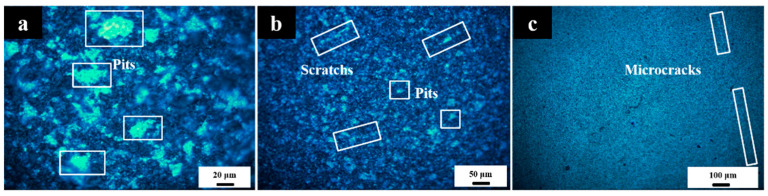
Crystal surface morphology after grinding with different abrasive particle size (**a**) 40 μm; (**b**) 20 μm; (**c**) 10 μm.

In the initial stage of ultra-precision machining of optical components, the grinding process is adopted. On the one hand, the shape and dimensional accuracy of crystal components are adjusted to meet the machining requirements; On the other hand, in order to remove the defect damage caused by the previous process, multiple grinding processes are usually used to remove layer by layer. By observing the surface morphology of sesquioxide crystals ground by abrasives with different particle sizes with a microscope, it can be seen that with the decrease of abrasive particle size, the surface damage of sesquioxide crystals, pits, and other surface defects decreases, and the defects in the form of microcracks increase. As shown in Figure 1, the abrasive particles from 40 μm (Figure 1a) were reduced to 20 μm (Figure 1b), and further reduced to 10 μm (Figure 1c). With the decrease in particle size, the macroscopic defects on the crystal surface decrease in both quantity and scale.

**Figure 2:**
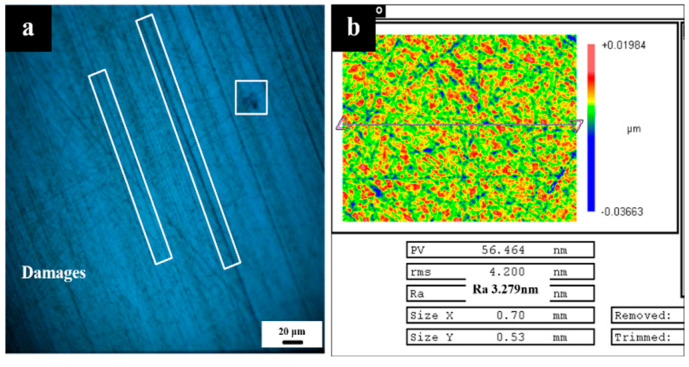
Crystal surface topography (**a**); and surface roughness (**b**) after pre-polishing.

In the grinding process, the abrasive particle size is reduced step by step. After grinding, there are still many small macro damages on the surface of the sesquioxide crystal in Figure 2a, which are difficult to remove Generally, the polished crystal components will use the pre-polishing process to quickly remove some defect layers of the crystal, so as to obtain better surface quality and provide good flatness for coupling the next processing process, as shown in Figure 2b.

Most of the defects on the crystal surface and sub-surface are produced by the above two processes. In the grinding and pre-polishing, abrasive with high hardness is used to remove and modify the surface. Under the action of normal pressure and tangential friction, micrometer defects and nanometer defects are produced on the surface. The above defects will be regarded as the key influencing factors to improve the surface quality in the next polishing process, and the improvement of surface shape needs to be taken into account. 

**Figure 3:**
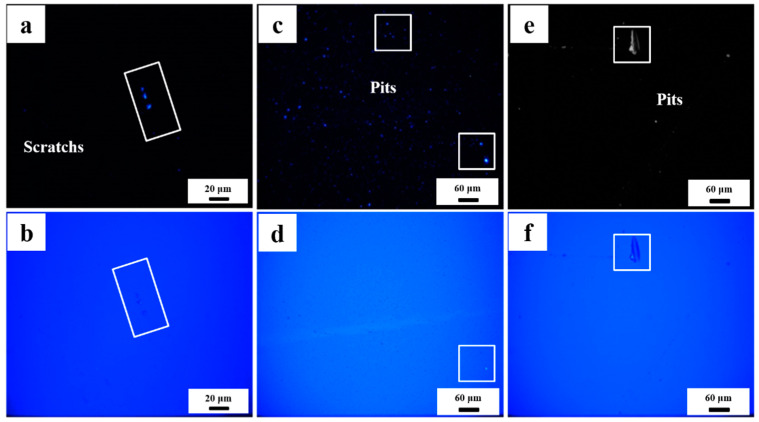
Scattered dark (**a**,**c**,**e**)/light (**b**,**d**,**f**) field imaging of crystal surface defects after fine polishing.

As shown in Figure 3, the macro defect morphology on the surface of the element is detected by scattering light/dark field imaging. Figure 3a–f are dark field and bright field images at the same position respectively. Although the fine polishing process removes a depth layer of about 3~5 microns on the surface in order to remove the defect layer of the previous process, there are still defects in the form of pit, scratch, and micro crack on the surface after fine polishing. These defects seriously reduce the service performance and service life of laser crystal elements, and further affect the operation stability of the laser system. Starting from the defect generation process and the influence of defects on the surface structure in the ultra-precision machining of laser crystal components, this paper is committed to providing a theoretical basis for the low defect machining of laser crystal surface through the research on the surface defect generation mechanism and material removal mechanism.

### 3.2. ReaxFF MD Simulation of Surface Defect Generation Process of Tm: GdScO_3_ Laser Crystal

In order to study the changes in the surface structure during mechanochemical wear, we used reactive force field (ReaxFF) for molecular dynamics simulation. Compared with other force fields, the reactive force field introduces the concept of bond order, takes the chemical bond ($BO_{ij}^{\sigma }$, $BO_{ij}^{\pi },\;BO_{ij}^{\pi \pi }$) as the function of $r_{ij}$, determines the connectivity of atoms according to the bond order of any two atoms in the simulation process, and simulates the fracture and formation of chemical bond [18,19,20,21,22,23,24,25,26,27]. The expression is shown in Formula 1:

(1) $$E_{system}=E_{bond}+E_{over}+E_{angle}+E_{tors}+E_{Coulomb}+E_{vaWaals}+E_{Specific}$$

where, $E_{system}$ is the total energy of the system, $E_{bond}$ is a continuous function of the interatomic distance which describe the energy associated with forming a bond between atoms, $E_{over}$ is the over coordination energy correction term which is based on the atomic valence rules. $E_{angle}$ and $E_{tors}$ are the energy associated with the valence angle strain and torsional angle strain. $E_{Coulomb}$ and $E_{vaWaals}$ are the Coulomb interaction and van der Waals interaction, respectively, $E_{Specific}$ refers to system-specific terms that are not normally included, such as lone pairs, conjugation, hydrogen bonding, and C_2_ correction.

The relationship between bond order and bond length is as follows:

(2) $$BO_{ij}^{\prime }=BO_{ij}^{\sigma }+BO_{ij}^{\pi }+BO_{ij}^{\pi \pi }=\exp\left[P_{bo1}\cdot {\left(\frac{r_{ij}}{r_{o}^{\sigma }}\right)}^{P_{bo2}}\right]+\exp\left[P_{bo3}\cdot {\left(\frac{r_{ij}}{r_{o}^{\pi }}\right)}^{P_{bo4}}\right]+\exp\left[P_{bo5}\cdot {\left(\frac{r_{ij}}{r_{o}^{\pi \pi }}\right)}^{P_{bo6}}\right]$$

where, $BO_{ij}^{\sigma }$, $BO_{ij}^{\pi }$ and $BO_{ij}^{\pi \pi }$ are the contribution of the σ, π and ππ bond to the bond order, respectively.$\;r_{ij}$ is the distance between *i* and *j* atoms. $r_{o}^{\sigma }$, $r_{o}^{\pi }$ and $r_{o}^{\pi \pi }$ are three different bond radii. $P_{boi}$ (*i* = 1, 2,..., 6) is a parameter fitted by experiment and experience. Therefore, the formation and splitting of keys can be determined by updating the key order in each calculation iteration step.

**Figure 4:**
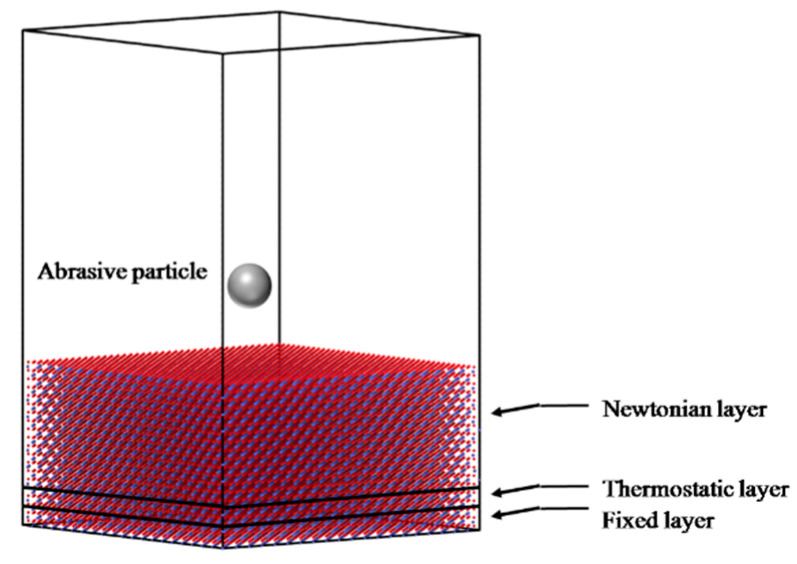
Schematic diagram of molecular dynamics model.

As shown in Figure 4, we have established a crystal model of 110.4 Å × 110.4 Å × 50.7 Å containing 48,001 atoms. The model is divided into three parts: Newtonian region, thermostat region, and frozen region. The atoms in the Newtonian region follow the Newtonian dynamic and are used to simulate the whole process. The Langevin thermostat is applied to the thermostat region to simulate the heat dissipation process and stabilize the temperature of the model. The atoms in the fixed region are completely fixed to support the whole model. Among them, the thickness of the thermostat region and frozen region are 5 Å and 8 Å, respectively. Spherical abrasive particles are added by the ‘fix indent’ command in large-scale atomic/molecular massively parallel simulation (LAMMPS). The diameter of the particles is 30 Å. Its interaction with the sample is described by the following formula:

(3) $$\begin{array}{r} F(r)=-K{\left(r-R\right)}^{2},\;r<R\\ F(r)=0,\;r>R \end{array}$$

where force constant *K* = 10 ev/Å^3^, r is the distance between the atom and the center of abrasive, and *R* is the radius of the abrasive.

All molecular dynamics simulations are carried out by LAMMPS. In each simulation, firstly, the conjugate gradient algorithm is used to relax the model to the minimum energy state. Then, the canonical ensemble (NVT ensemble) is used to balance the model for 50 ps at the temperature of 300 K to minimize the internal energy of the model and make it reach the equilibrium state. In the process of mechanical wear, NVE ensemble (microcanonical ensemble) is used in the Newtonian region and the NVT ensemble combined with the Langevin thermostat is used in the constant temperature region. The time step is set to 1 fs to meet the requirements of the ReaxFF potential function, periodic boundary conditions are used in all directions of the structure, and a vacuum layer with a thickness of 150 Å is added in the Z direction to avoid boundary effect. The abrasive particles are vertically pressed into the sample at a speed of 2 Å/ps above the sample, and then vertically removed at the same speed. For mechanically ground samples, first press the particles vertically into the sample, then apply lateral motion at a speed of 2 Å/ps to produce shear force, and finally separate the particles vertically.

**Figure 5:**
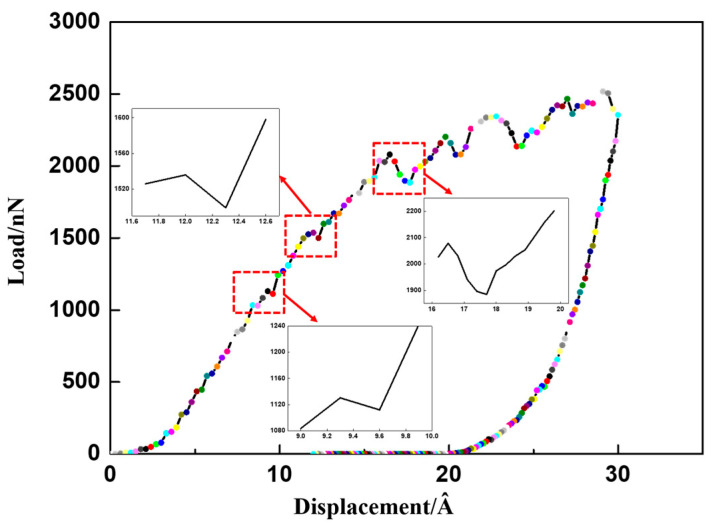
The evolution of the indentation force with the indentation depth during indentation at the loading-unloading rate of 2 Å/ps.

Figure 5 shows the change of load with displacement when the abrasive particles approach the substrate surface at a moving speed of 2 Å/ps. The applied load shows several drops in the figure. The first load drop is located at the displacement of 9.3~9.9 Å, which is also known as the ejection effect, which is related to the initial plasticity of the base material. The second load drop is located at the displacement of 11.7~12.3 Å, which is attributed to the maximization of the amorphous layer. The third load drop is at 16.2~18.3 Å, which may be due to the sudden increase of distortion layer.

Through the trace diagram of molecules in the whole process, the generation process of the changes in crystal surface and sub-surface structures in the ultra-precision machining process is studied. In the grinding process, the force between the abrasive particles and the surface of the component is divided into two stages, the first is a normal pressure, and then the normal pressure and tangential friction exist at the same time. In this paper, the two stress processes are simulated respectively.

**Figure 6:**
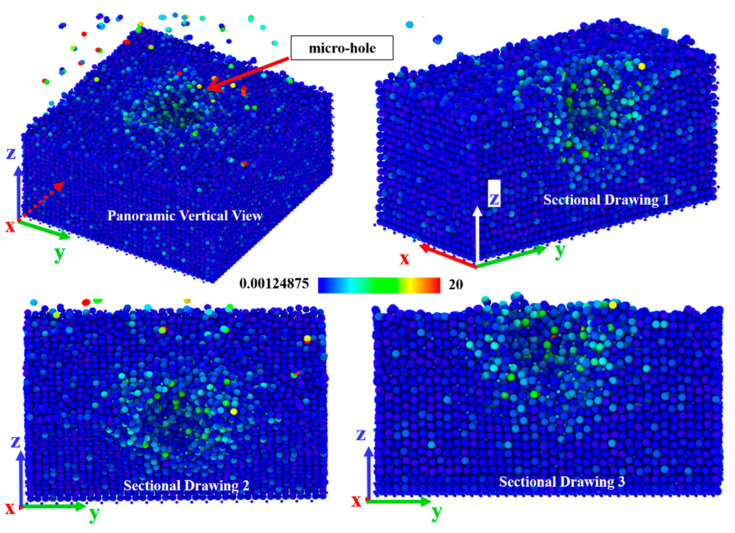
The panoramic vertical view and sectional drawings of structure changes when the abrasive particles have only normal pressure on the surface.

As shown in Figure 6, when the abrasive particles only have normal pressure on the surface of the element, the atoms at the stress position on the crystal surface have been displaced, and micro surface defects (atomic peeling/displacement) will be observed. ReaxFF MD simulation shows that at this time, because there is no shear effect caused by tangential friction, the atomic displacement basically occurs in the z-axis direction, and the directly stressed position. Except for the atoms removed from the structure, the rest of the stressed atomic structure is compressed, and the original lattice structure is deformed, which shows that there are micro “holes” on the material surface.

**Figure 7:**
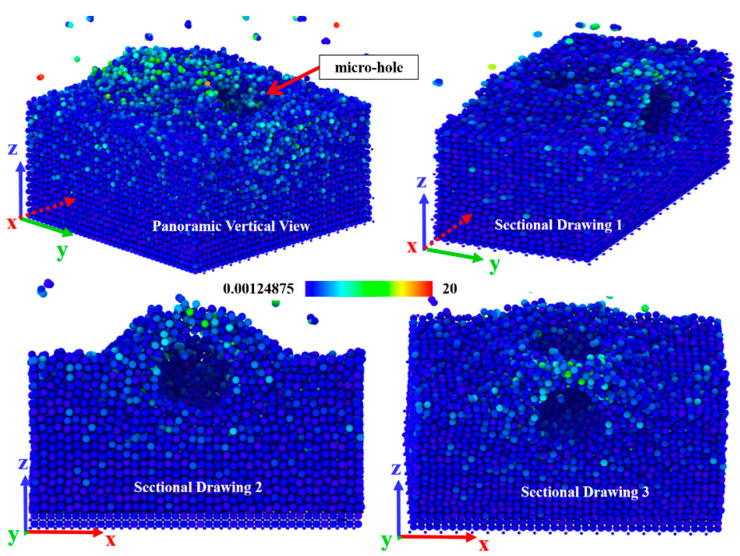
The panoramic vertical view and sectional drawings of structure changes when the abrasive particle have both normal pressure and tangential friction on the surface.

When the abrasive particles have both normal pressure and tangential friction on the surface of components, this situation is closer to the real machining process. It is equivalent to the abrasive particles pressing the surface of the element forward and rolling on the surface. At this time, the surface atoms are not only under normal pressure, but also under the action of tangential friction. At the same time, the sub surface atoms will feel the shear stress along the sliding direction, because they are covalently bonded with the outermost atoms. ReaxFF MD simulation shows that at Figure 7, the atoms in the outermost layer have peeled off and bulged (about to peel off), the atomic structure in the bulge has become loose, while the atoms in the sub surface layer have shifted, resulting in the distortion of the original structure. Different from the previous case, at this time, the atomic displacement occurs on both z-axis and x-axis. In this case, micro “scratches” appear on the material surface.

**Figure 8:**
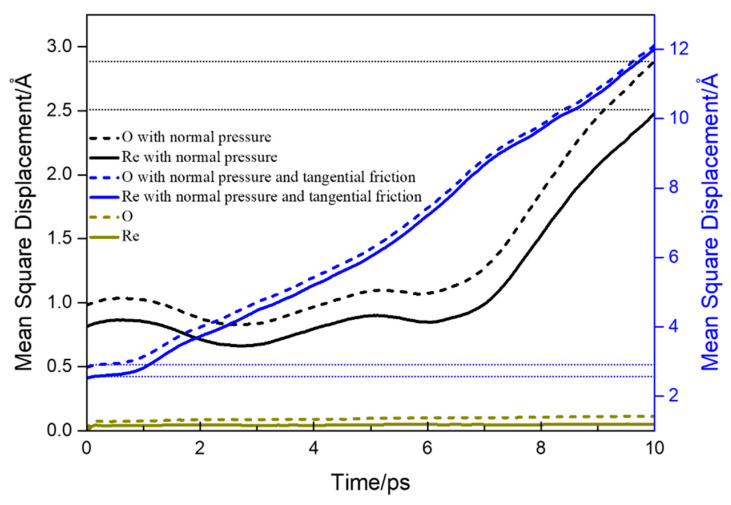
The mean square displacement of system atoms under different contact conditions.

Based on the calculation of mean square displacement (MSD) in MD simulation, we calculated the dynamic tendency of a single atom during friction, as shown in Figure 8. Compared with the stable Re_2_O_3_ crystal system, the mean square displacement (MSD) of atoms in the system increases rapidly whether the surface is only subjected to normal pressure or both normal pressure and tangential friction, in Figure 8. In the presence of tangential friction, the diffusion of atoms in the system becomes particularly intensified, which is affected by the increase of system temperature caused by friction heat generation. The increase of thermal diffusion leads to strong distortion of surface and subsurface lattice, which is also proved in the next chapter. At the same time, it can be found that compared with the stable and orderly bulk material structure, the sharp increase of MSD after friction and extrusion indicates that the surface structure becomes loose and disordered. According to Hertz contact theory, if the contact deformation is elastic, no material loss is expected [27]. However, our data show that even if the contact pressure (as shown in Figure 5) is much smaller than the bulk material elastic modulus (197.21 GPa) and shear modulus (80.85 GPa, the data were measured by the UMS-200 advanced TECLAB ultrasonic echo material characterization system), the surface of the base material will still have micro defects due to structural distortion.

In the next chapter, firstly, the relationship between polishing pressure and macro defects is studied through single factor grinding and polishing experiments, and the influence of abrasive hardness is considered (abrasive hardness will affect the action component of polishing pressure on the surface). However, due to the lack of experimental means and theoretical cognition, it is difficult to establish a direct relationship between such micro defects and macro defects. Therefore, through the nano-scratch experiment, we characterized the manifestation of macro defects on the micro scale.

### 3.3. Generation Mechanism of Surface Defects of Tm: GdScO_3_ Laser Crystal

In the previous chapter, a single particle abrasive removal mechanism model was established. It was found that the atoms on the surface layer of the substrate peeled off and shifted under the action of the normal pressure and tangential friction of the abrasive. In order to control surface/sub surface defects and obtain optical elements with high resistance to laser damage, in the previous laser crystal processing process, it is usually hoped to gently remove the defects caused by the previous process without introducing new defects by reducing the abrasive pressure, hardness and particle size [28,29]. In this simulation, even if the contact pressure is much smaller than the bulk material elastic modulus and shear modulus, micro defects will still occur on the surface/sub surface of the substrate. Next, we will discuss the conclusion of this model from the perspective of macro damage and micro defect.

(1) Effect of load on macroscopic surface defects.

**Figure 9:**
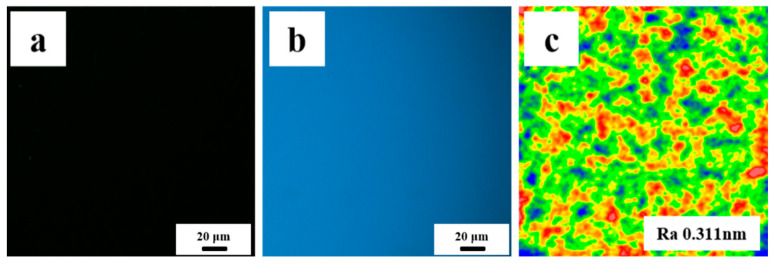
Initial surface quality of samples (**a**) dark field image; (**b**) bright field image; and (**c**) typical roughness distribution diagram.

**Figure 10:**
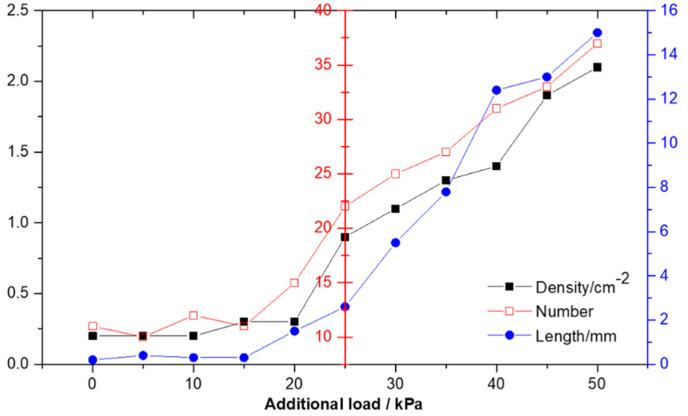
Statistical results of surface scratches under different processing pressures.

**Figure 11:**
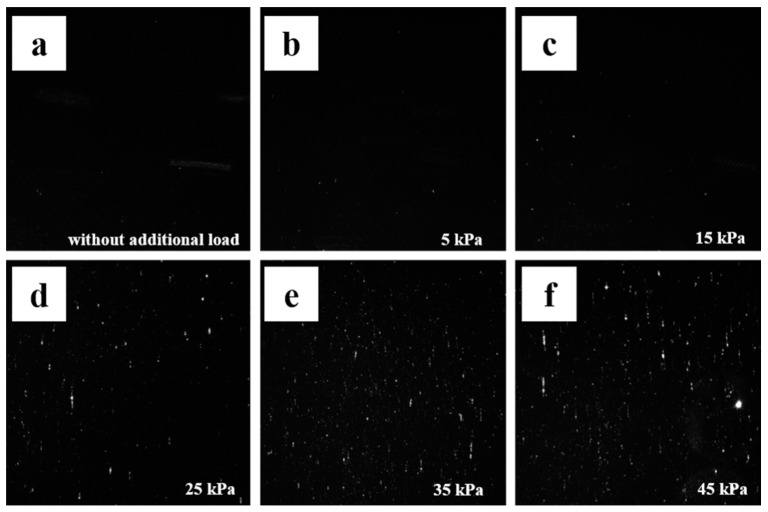
Typical defect distribution diagram of components under different additional loads (**a**) zero load; (**b**) 5 kPa; (**c**) 15 kPa; (**d**) 25 kPa; (**e**) 35 kPa; (**f**) 45 kPa.

We select Tm: GdScO_3_ laser crystal (size Φ 30 mm × 2 mm, elastic modulus 197.21 GPa, shear modulus 80.85 GPa, static fracture toughness 0.94 Mpa m^1/2^. The data were measured by the UMS-200 advanced TECLAB ultrasonic echo material characterization system) as test samples. The initial surface quality of the sample is good, the surface has no obvious macroscopic defects, and the average surface roughness is 0.3 nm, as follows in Figure 9:

The real grinding and polishing process is closer to the second case in the model. The existence of a normal load is bound to be accompanied by tangential friction. Thus, we choose to simplify the real processing conditions, carry out grinding and polishing on the CCOS machine tool, fix other process parameters, adjust the polishing pressure, and study the impact of load on surface defects. The statistical results of surface defects under different processing pressures are shown in Figure 10 and Figure 11.

The influence of polishing pressure on surface defects is analyzed through the experimental results (medium hardness polyurethane polishing disc, revolution speed of 50 r/min, particle size of 0.1 μm). It can be seen from Figure 11 that with the decrease of polishing pressure, the surface damage of components begins to improve obviously, but when it decreases to about 15 kPa, the number and length of damage points will no longer decrease, but fluctuate in a small range and tend to be stable, as shown in Figure 10. It is possible that when the pressure is higher than 15 kpa, the coarser abrasive will be pressed into the grinding disc at a greater depth and turn into two-body grinding behavior, resulting in scratches on the surface micro cutting process. When the loading pressure is reduced to 15 kpa, the number of effective Abrasives between the polishing disc and the element surface decreases by a certain value and remains stable. Even if the load pressure is further reduced, the change of tangential resistance and friction coefficient is very small, so the continuous reduction of load at this time cannot significantly improve the surface quality. In the actual processing process, because the removal efficiency at this time is very low, and the residual defects of the previous process cannot be effectively removed, the generation of surface defects can only be controlled to a certain extent by reducing the load. Such experimental results verify the MD simulation to some extent. By reducing the processing pressure, surface defects cannot be completely avoided.

(2) Effect of abrasive hardness on surface defects.

In the actual machining process, the polishing pressure does not directly act on the component surface, but applies a load on the component surface with the help of the intermediate abrasive particles. Therefore, the hardness of the abrasive will also affect the payload on the surface of the component. Generally, the greater the hardness of the abrasive, the higher the removal efficiency. However, the greater the hardness of the abrasive, the greater the depth of the concave layer on the surface of the component, and the concave depth increases with the increase of the particle size. In the process of precision polishing of optical elements, abrasives with hardness close to or slightly lower than that of the substrate will be selected under normal circumstances, so that the effective removal amount can be guaranteed without causing obvious deterioration of surface quality. In order to verify the influence of abrasive hardness on the surface quality of components, CeO_2_, SiO_2_, Al_2_O_3_, and diamond powder with increasing hardness are used as abrasives to grind and polish the surface of crystal components.

**Figure 12:**
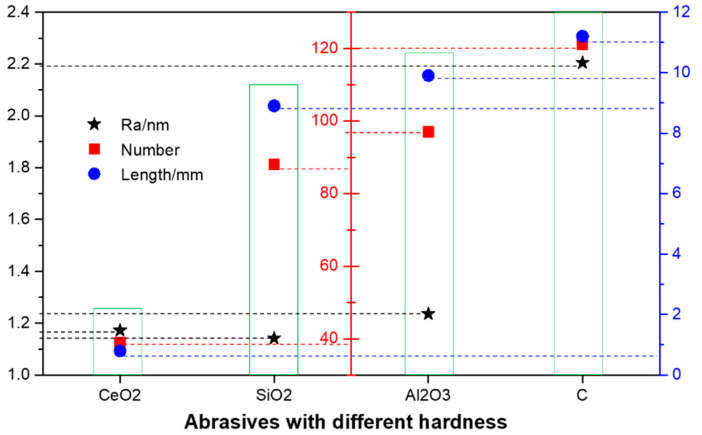
Statistical results of component surface scratch and average roughness after abrasive machining with different hardness.

**Figure 13:**
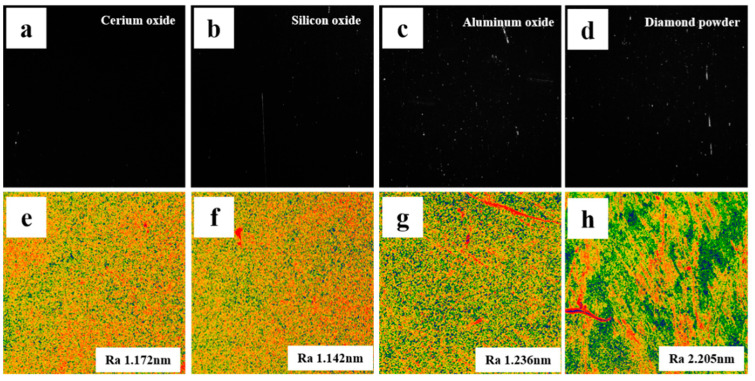
Typical defect and roughness distribution diagram of the component surface under the action of abrasive with different hardness (**a**) CeO_2_ and (**e**) Ra 1.172 nm; (**b**) SiO_2_ and (**f**) Ra 1.142 nm; (**c**) Al_2_O_3_ and (**g**) Ra 1.236 nm; (**d**) C and (**h**) Ra 2.205 nm.

Figure 12 and Figure 13 show the effect of abrasive with a different hardness on surface defects and distribution (soft polyurethane polishing disc, abrasive particle size is 1 μm, revolution speed 50 r/min, polishing pressure 20 kPa). It can be seen that the surface of components grinded with diamond powder and Al_2_O_3_, which are much harder than Tm: GdScO_3_ crystal, is densely scratched. There are still many shallow and fine scratches on the surface after grinding with SiO_2_ which is close to the hardness of the base material, and the total length is not significantly reduced compared with that before. However, the surface polished with soft CeO_2_ showed fewer micron scale scratches under the microscope. During the grinding process, the larger self-deformation of CeO_2_ particles relative to diamond powder and Al_2_O_3_ abrasive under the same pressure offsets part of the abrasive cutting depth, so the amount of scratches is small. However, the surface roughness of the components was observed by the white light profiler. The surface roughness of the diamond powder with the largest hardness after grinding was the worst. The roughness of the surface of CeO_2_, SiO_2,_ and Al_2_O_3_ after grinding had little difference, floating up and down by about 7%.

Experience shows that the use of soft Abrasives can improve the surface quality, but we find that it only plays a certain role in the improvement of micron scale defects, and nano-scale and even atomic scale defects have not been reduced. Although the micro scale surface defects are much smaller than the macro micron scale surface defects, for the laser optical elements that need to be used in the high-power state, it will reduce their ability to resist laser damage and affect the stability of the system.

However, it should be pointed out that molecular dynamics simulation is based on the nano-scale, which can better describe the micromechanical behaviors such as crystal defects (phase transition, crystallization and dislocation) and material deformation in the nano-cutting process. In MD simulation, the boundary conditions are fixed, and the defects are micro atomic scale. At the same time, the defects in the actual machining process are macro scale, and even if the variables are controlled, there are still many uncontrollable factors. Due to the lack of representation means and theoretical cognition, although experts in the field have conducted much research on the removal mechanism of atomic scale in ultra-precision machining, the mapping relationship between molecular dynamics models and macro phenomena is not clear, which has also become the biggest obstacle for many models based on the development of an atomic scale to directly guide the actual machining process.

Therefore, in the next section, the composition of the surface defect layer is further explored by characterizing the microstructure changes at the atomic/molecular scale at the scratch location. The results of the nano-scratch experiment and the structure change prediction of the molecular dynamics model are mutually confirmed.

(3) Effect of abrasive on molecular scale surface/subsurface defects

**Figure 14:**
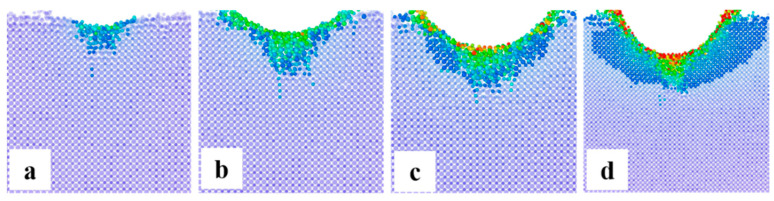
Center cross-section views of structural evolution at the indentation depth of (**a**) 3.0 Å, (**b**) 6.0 Å, (**c**) 10.5 Å and (**d**) 18.0 Å.

As shown in Figure 14, we discussed the structural amorphization and its effect on the surface structure. The amorphization starts directly below the abrasive particles, and the obvious amorphization layer and distortion layer begin to appear at the depth of 3.0 Å. With the increase of indentation depth, the distortion layer becomes wider gradually, but the amorphous layer does not increase further with the increase of indentation depth after it is stable at 10.5 Å. The reason may be that the outer atoms bear pressure directly, and the high compressive stress caused by the increased loading makes the amorphous region expand and thin along the contact surface.

Although the molecular dynamics model predicts that the structure will change after the abrasive presses the crystal surface, due to the size of the model, it is impossible to directly predict the thickness and detailed structure of the defect layer. In order to further study the microscale structure changes of substrate materials caused by abrasive under pressure, a nano-scratch instrument was used to generate scratches on the crystal surface, and the structure of the scratch interface was analyzed.

**Figure 15:**
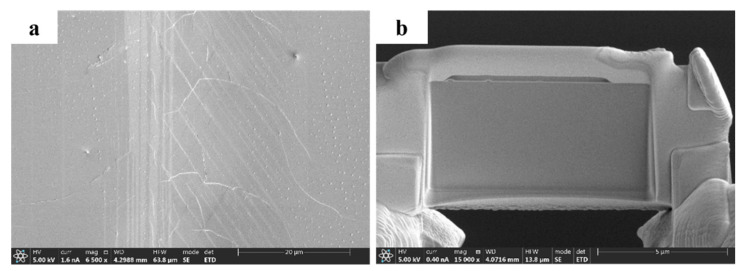
Sample at low magnification (**a**) part-graph and (**b**) panoramic drawings of samples.

Using a spherical grinding head, scratches were generated on the crystal surface under the condition of constant normal load and tangential friction, and observed under a low power microscope, as shown in Figure 15a. At low magnification, it can be found that in addition to the main scratch direction, there are some tree-shaped cracks spreading around, which may be caused by repeated extrusion and friction at the same position. Due to the brittleness of the Tm: GdScO_3_ laser crystal, under the normal phase load far less than the elastic modulus of the crystal, the tree crack around the main scratch also proves that the atoms in the direct compression area fall off, and cause the obvious displacement of nearby atoms, resulting in the appearance of dendritic brittle crack.

In order to further observe the phase change inside the scratch, FIB (Focused Ion beam) was used to obtain samples for HRTEM (high-resolution transmission electron microscopy) to observe, as shown in Figure 15b.

**Figure 16:**
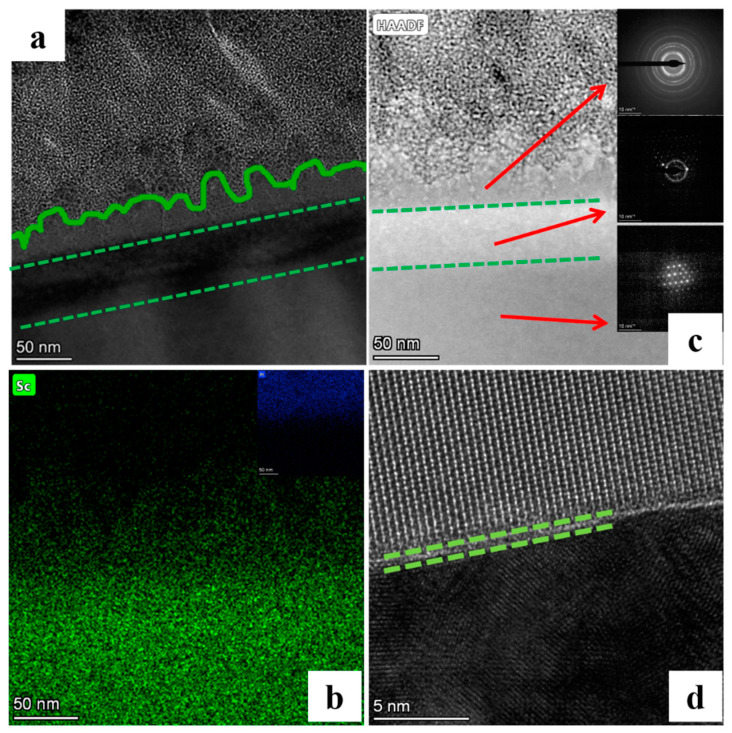
High-resolution transmission electron microscopy of surface defects/matrix micro region (**a**) TEM, (**b**) HAADF image with electron diffraction pattern of three regions, (**c**) EDS map, and (**d**) HRTEM.

As shown in Figure 16a, it can be seen from the HTEM image of the scratch position that the scratch layer (direct stress layer) presents a concave-convex structure (green curve), which has an obvious boundary with the sub surface layer, and the sub surface layer is unevenly distributed due to its proximity to the stress layer. As shown in Figure 16b, mapping scanning can find that the atoms of the concave convex scratch layer are loosely arranged relative to the matrix atoms and gradually become disordered. As shown in Figure 16c, three states of the scratch damage layer can be found by mapping scanning: the atomic arrangement of the directly stressed layer is distorted, and the diffraction image shows amorphous information; in the sub surface layer structure, both crystalline and amorphous exist, while the substrate structure still retains the original complete crystal structure. We believe that the scratch damage layer is composed of an amorphous layer with a thickness of 40–50 nm, a distortion layer with a thickness of 50–60 nm, and a substrate layer. As shown in Figure 16d, the HRTEM image of the distortion layer shows that there are many defects in the structure, including stacking faults, torsion, and dislocations. This may be because it is close to the stress layer, and the atomic displacement of the stress layer drives the synchronous displacement of the sub surface layer. In addition to brittle cracks, although the phenomenon of atomic peeling in the sub surface layer is very rare, many large-depth atomic displacements directly cause the phase change of the layer. This will be reflected in the change of micro mechanical properties of the material, and may also make the use effect of the material fail to reach the ideal state that the base material should perform to a certain extent.

## 4. Conclusions

This work shows the formation and deformation of defect layer structure on the surface of substrate material during ultra-precision machining of sesquioxide laser crystal GdScO_3_ studied by molecular dynamics simulation. The main conclusions are as follows:

(1) The atom displacement diagram of the simulation results shows that under the action of minimal normal phase load and tangential friction, the surface atoms of the substrate material peel off and shift. This combination of atomic spalling and displacement at the micro scale is considered to constitute the removal of materials at the macro level.

(2) With the increase of indentation depth, under the action of surface load, the amorphous layer appears outside the defect structure and becomes stable, and the structure distortion appears in the secondary layer and increases with the development of displacement.

(3) It is proved by experiments that reducing the machining pressure and using soft abrasives can only improve the macro surface quality in a certain range, but the micro defects on the material surface are difficult to avoid. However, the mapping relationship between micro defects and macro damage is limited by the current experimental conditions and theoretical understanding level, and it is still unable to establish a direct relationship, which needs to be further explored.

(4) Through the structural characterization of macro defects, it is found that the micro defect layer with a thickness of 100 nm is composed of an amorphous layer, distortion layer, and substrate layer after the abrasive particles act on the crystal surface under normal pressure.

Micro defects may not directly show as macro damage, but due to the change of crystal structure, it will affect the laser damage resistance and service life of high-power laser optical elements used under high-power conditions, and may affect the stable operation of the system. In the ultra-precision machining process, there are many studies on the control of surface/sub surface macro defects of optical elements, the formation mechanism, and control technology of surface micro defects also need more attention.

## Data Availability

Not applicable.
